# Antibody-Drug Conjugates: A Promising Novel Therapy for the Treatment of Ovarian Cancer

**DOI:** 10.3390/cancers12082223

**Published:** 2020-08-09

**Authors:** Aranzazu Manzano, Alberto Ocaña

**Affiliations:** Medical Oncology Department, Experimental Therapeutics Unit, Hospital Clínico San Carlos, 28040 Madrid, Spain

**Keywords:** antibody-drug conjugate, ovarian cancer, cleavable linker, non-cleavable linker, bystander effect, payload

## Abstract

Antibody-drug conjugates (ADCs) represent a novel and promising therapeutic strategy for the treatment of cancer patients. ADCs target antigens highly expressed on the membrane surface of tumor cells to selectively deliver a cytotoxic drug. Ovarian tumors differentially express tumor-specific antigens, which can be used to guide ADCs. This strategy allows for optimizing tumor targeting while minimizing systemic toxicity compared to classical chemotherapeutic agents. ADCs can be improved by using a cleavable linker allowing the delivery of the toxic payload in surrounding cells not expressing the target protein, therefore acting on heterogeneous tumors with different cell populations. Currently, more than 15 ADCs are under preclinical investigation in ovarian cancer, and some of them have already been tested in early-phase clinical trials with promising results. In this review, we summarize the mechanism of action and the toxicity profile of ADCs and discuss the latest preclinical discoveries and forthcoming applications in ovarian cancer.

## 1. Introduction

Ovarian cancer is one of the most lethal cancers in women. The initial standard of care treatment includes surgery (upfront or cytoreductive interval debulking surgery) and platinum-based chemotherapy [1]. Bevacizumab (antibody targeting vascular endothelial growth factor) and PARP (poly-ADP-ribose polymerase) inhibitors are the only targeted therapies currently approved for the treatment of this disease [2,3]. Although these agents have changed the prognosis of some patients, the majority of them will still recur and unfortunately succumb to this disease. New approaches are urgently needed to improve outcomes in this malignancy.

Over the last three decades, antibodies have emerged as a good therapeutic option for the treatment of cancer. By binding specific antigens on the tumor cell membranes, they can mediate the inactivation of the downstream oncogenic pathway in addition to induction of an immune response [4]. The conjugation of an antigen-targeting antibody with a cytotoxic drug allows selective delivery of the drug to antigen-expressing cells, increasing efficacy and decreasing systemic toxicity in comparison to classic chemotherapeutic agents [5]. In the last decade, eight antibody-drug conjugates (ADCs) have been approved for the treatment of hematologic malignancies (gemtuzumab ozogamicin, brentuximab vedotin, inotuzumab ozogamicin, and polatuzumab vedotin-piiq), HER2-expressing breast cancer tumors (aldo-trastuzumab emtansine and trastuzumab deruxtecan), triple-negative breast cancer (sacituzumab govitecan) and urothelial carcinomas (enfortumab vedotin), and other compounds are being investigated in different solid tumors, including ovarian cancer [6,7]. Ovarian tumors differentially express a great number of tumor-antigens that can be used for this novel strategy [8]. In this review, we summarize the mechanism of action, resistance, and toxicity profile of ADCs and their current status in ovarian cancer.

## 2. Structure of ADCs and Toxicity Profile

An antibody-drug conjugate (ADC) consists of an antibody specifically directed against a tumor-antigen, which is conjugated through a molecular linker with a cytotoxic agent, that ultimately will be internalized and released into tumor cells [5]. The three components of ADCs (antibody, toxic payload, and linker) are crucial for both efficacy and toxicity.

### 2.1. Antibody/Antigen

The antibody included in an ADC is usually a humanized immunoglobulin G engineered to attach the toxic payload to certain residues of the antigen-binding regions (Fabs) [9]. The tumor-antigen selected should be preferentially expressed on the membrane surface of tumor cells and minimally expressed in normal tissues, in order to minimize systemic toxicity. Antibody-antigen binding triggers internalization by endocytosis and lysosomal degradation of the complex, delivering the cytotoxic payload into tumor cells [10,11]. Folate receptor alpha, mesothelin, MUC16, TROP2, tissue factor, and NaPi2b are common antigens used for conjugation in this malignancy, as they are usually overexpressed in epithelial ovarian cancer [12]. Selecting the correct antigen is a crucial step for effectiveness, and many efforts have been developed to identify novel antigens. Table 1 shows a complete list of antigens used as targets for the development of ADCs that are under evaluation in ovarian cancer, including those in clinical and preclinical stages.

### 2.2. Payload

As only 1% of the administered ADC reaches the target tumor site, the ideal payload should be small molecules with potent activity, usually at picomolar range, with direct cytotoxic effects and a high therapeutic index [28]. Few molecules to date have been identified as optimal payload candidates for conjugation processes. Among them, the most commonly used for ovarian cancer ADCs are the two microtubule inhibitors monomethyl auristatin (MMAE/vedotin) and DM4 (ravtansine/soravtansine) [10,11]. Conjugation with the antibody enables the specificity of these drugs to be increased as well as reducing systemic toxicity. The payload delivery to targeted cells is limited by antigen expression and the average of drug molecules conjugated to the antibody, the so-called drug-to-antibody ratio (DAR). Overloading ADCs with lipophilic payloads causes aggregation, leading to an increased hepatic uptake and potential systemic toxicity risk [29].

### 2.3. Linker

The linker is a chemical structure that binds the antigen-targeting antibody to the cytotoxic payload. Ideally, it should maintain this binding stable and unaltered through the bloodstream and release the drug only after antigen-antibody binding in antigen-expressing cells [30]. According to their chemical characteristics, linkers can be cleavable or non-cleavable. Cleavable linkers are chemically labile structures that can be cleaved depending on certain intracellular circumstances such as acid pH levels, high levels of glutathione, or the action of lysosomal proteases [31]. Some cleavable linkers can also deliver the drug extracellularly, i.e., in the acid pH tumoral microenvironment, inducing killing in nearby tumor cells with no expression of the targeted antigen. This bystander killing is an attractive effect for heterogeneous tumors in which not all tumor cells express the selected antigen and it depends on the permeability profile of the released payload [32]. Non-cleavable linkers release the drug only when the antibody is internalized and degraded inside the lysosome of the targeted cell. A non-specific killing of surrounding tumor cells is also possible as a result of the cytotoxic payload release from the apoptotic targeted-tumor cell [33].

### 2.4. Toxicity Profile

In global terms, ADCs have a favorable toxicity profile with low grade and easily manageable side effects [34]. On-target toxicity affecting non-tumor cells expressing the target antigen can be observed. The type and severity of this kind of toxicity will depend on the tissues where the antigen is expressed and the intensity of the expression itself [35]. Some characteristic off-target toxicity has also been described in phase 1 and 2 trials as a result of an early payload release into the systemic bloodstream. For ADCs containing microtubule cytotoxic agents, such as MMAE or DM4, significant hematologic toxicity can reach up to 5% [36]. In vitro studies have proposed cytotoxic damage by the released payload into hematopoietic stem cells of the bone marrow as a potential mechanism for this toxicity [37]. Grade 3–4 hepatic impairment, gastrointestinal toxicity, mainly diarrhea, and peripheral neuropathy are also frequently reported [36,38]. Ocular toxicity is a known class effect of ADCs and dose-limiting toxicity in many phase 1 trials. Reversible blurry vision and keratopathy are frequent and usually non-severe among patients under this therapy and can be managed with dose adjustments or treatment delays [39]. Primary prophylactic use of corticosteroid eye drops could be useful to avoid dose modification [40]. The mechanisms of corneal damage associated with ADC treatments are still unclear. It is unlikely to be an on-target effect as the majority of the antigens targeted by the ADCs are not significantly overexpressed in the eye, except for MUC16-targeted ADCs [41]. Some authors have postulated damage to stem cells located in the cornea as a possible causative mechanism [39].

## 3. ADCs in Ovarian Cancer

To date, there are no ADCs approved for the treatment of ovarian cancer. While only one molecule has achieved a phase 3 trial with encouraging data mainly in combination with chemotherapy or targeted therapy, the majority of ADCs are still in early development with different results (Table 2 and Table 3). Here, we review the main efficacy data in phase 1–3 trials.

### 3.1. Anti-Folate Receptor Alpha-Based ADCs

Folate receptor alpha (FRα) mediates folate uptake into cells, which is needed for DNA synthesis, cellular metabolism, and proliferation, and it is marginally expressed in normal cells [47]. In contrast, it is overexpressed in up to 90–95% of epithelial ovarian carcinomas, mainly in serous and endometrioid subtypes [48]. Mirvetuximab soravtansine (ImmunoGen Inc., Waltham, MA, USA) is an anti-FRα ADC conjugated with the tubulin-targeting DM4 through a cleavable linker, with promising activity in epithelial ovarian carcinoma. The overall response rate (ORR) in a phase 1 escalation cohort of platinum-resistant ovarian cancer (*n* = 44) was 26%, with a median progression-free survival (mPFS) of 4.8 months. The recommended phase 2 dose (RP2D) was established at 6 mg/kg intravenously adjusted to ideal body weight to reduce ocular toxicity [13,14]. After these encouraging results in an initially unselected population, the phase 3 FORWARD I trial (ClinicalTrials.gov Identifier: NCT02631876) compared mirvetuximab soravtansine to chemotherapy according to the investigator’s choice (weekly paclitaxel, pegylated liposomal doxorubicin or topotecan) in platinum-resistant ovarian cancer with ≥50% of FRα expression assessed by the 10× method (percentage of stained cells in ≥10× magnification by immunochemistry). Preliminary results were communicated at the 2019 European Society of Medical Oncology Congress (ESMO2019) with no differences in PFS, the primary endpoint of the trial, for the entire global population (HR 0.981, *p* = 0.89). In the pre-specified subgroup of high FRα expression (≥75%), the median PFS was slightly better for the ADC compared to chemotherapy (4.8 months vs. 3.3 months, HR 0.69, *p* = 0.049), with a trend towards a better overall survival (OS) with still immature data (16.4 months vs. 12 months, HR = 0.67, *p* = 0.048). In an exploratory analysis of FORWARD I using the PS2 method to assess FRα positivity (intensity of staining plus percentage of positively stained tumor cells), a higher ORR (26% vs. 6%) and higher PFS (5.6 months vs. 3.2 months, HR 0.54) were confirmed [15,16]. The correct method for scoring FRα expression remains unclear and will be of key importance for the future development of this and other ADCs.

The combination of mirvetuximab soravtansine with chemotherapy or targeted therapies seems to offer better results in the ongoing multicohort phase 1b/2 trial FORWARD II (ClinicalTrials.gov Identifier: NCT02606305). Combination with bevacizumab in heavily pretreated platinum-resistant ovarian cancer patients offers interesting results in a recently published phase 1b trial, with an ORR of 39% including five complete responses. The activity of the combination was higher in bevacizumab naïve patients and medium-high FRα expression by immunochemistry (ORR 56%, mPFS 9.9 months) [17]. The activity of mirvetuximab soravtansine has been also evaluated in platinum-sensitive ovarian cancer with at least 25% FRα staining in combination with carboplatin AUC4-5 in another phase 1b trial. The combination was well-tolerated, with fatigue, gastrointestinal symptoms, and blurred vision as the main side effects, an ORR of 71% (3 complete response (CR) and 9 partial response (PR)), and 15 months of median PFS [18]. The combination with immunotherapy has been also explored, with encouraging activity with some long-lasting responses [49].

Other anti-FRα ADCs under investigation in phase 1 trials are STRO-002 (SutroBiopharma Inc., San Francisco, CA, USA) and MORAb-202 (Eisai Inc., Tokyo, Japan). STRO-002 is composed of SP8166 (H01), an FRα human immunoglobulin G1 (IgG1) antibody, conjugated to a proprietary cleavable drug linker, SC239, containing a tubulin-targeting payload. The STRO-002-GMI phase 1 trial in unselected ovarian and endometrial cancer patients is still enrolling with the first patient included in March 2019 and is pending results [42]. MORAb-202 consists of farletuzumab (a humanized monoclonal antibody that binds to FRα conjugated to eribulin mesylate through a cleavable linker). The phase 1 dose-escalation trial in FRα positive solid tumors has shown promising data with 75% disease control rate (DCR) for the entire population, including 1 CR and 2 PR among the 9 patients with ovarian cancer included in the trial [43].

### 3.2. Anti-NaPi2B-Based ADCs

NaPi2B is a sodium-dependent cell-surface transporter normally expressed in lung and small intestine epithelial cells [50]. High expression of this protein can be observed in serous ovarian tumor cells compared to non-malignant ovarian cells [51]. Lifastuzumab vedotin (LIFA, Genetech Inc., San Francisco, CA, USA) is an antiNAPi2B ADC conjugated with MMAE with a protease-cleavable linker. The activity of LIFA has been assessed in a phase 2 trial in unselected platinum-resistant ovarian cancer patients (*n* = 99) compared with standard pegylated liposomal doxorrubicin (PLD). Median PFS was 5.3 months vs. 3.1 months (HR 0.71), favoring ADC without differences according to NaPi2B expression. ORR was also higher (34% vs. 15%, *p* = 0.03) in patients treated with LIFA. Neuropathy was more frequently observed in the experimental arm (11% vs. 4%) [19].

XMT1536 (Mersana Therapeutics, Cambridge, MA, USA) is another antiNAPi2B ADC with an auristatin payload conjugated through a cleavable linker. The chemical structure through its fleximer polymer linker allows a higher DAR (10–12), which could be translated into higher efficacy. Interim data from the phase 1 trial presented at the American Society of Clinical Oncology Congress (ASCO 2019 and ASCO 2020) showed 2 CR and 11 prolonged stable disease in platinum-resistant ovarian cancer without significant adverse effects [46].

### 3.3. Anti-MUC16-Based ADCs

MUC16 is the transmembrane portion of the CA125 antigen, typically overexpressed in epithelial ovarian cancer cells [52]. DMU4C064A (Genetech Inc.) containing MMAE payload ADC has shown interesting results in a phase 1 trial, with 45% ORR including 1 CR and 8 PR with a mPFS of 5.8 months. Ocular toxicity was frequent, affecting up to 75% of patients [45]. Another MMAE-containing anti-MUC16 ADC with a protease-cleavable linker, sofituzumab vedotin (DMUC5754A, Genentech, Inc.), has shown modest results (ORR 17%), with no further development [20].

### 3.4. Anti-Mesothelin-Based ADCs

Mesothelin is a glycoprotein that covers different corporal cavities (i.e., pleural or peritoneum) participating in cell adhesion. It is overexpressed in 70–85% of epithelial ovarian carcinomas [53]. Anetumab ravtansine (BAY 94-9343, Bayer AG, Leverkusen, Germany), a DM4-containing ADC with a cleavable linker, has shown robust activity in combination with PLD in preclinical studies. A phase 1b trial in combination with PLD was conducted in platinum-resistant disease, offering durable responses with a disease control rate (DCR) of 83% with 52% PR (11/21) and 33% stable disease (7/21) [21]. A combination phase 2 trial with bevacizumab compared to paclitaxel in refractory ovarian cancer is currently ongoing (ClinicalTrials.gov Identifier: NCT03587311).

DMOT4039A (RG-7600, Genetech Inc.) is another anti-mesothelin MMAE-containing ADC. Results of a phase 1 trial in unresectable pancreatic (*n* = 40) and platinum-resistant ovarian cancer (*n* = 31) have shown disappointing results with only 4 confirmed PR [44], resulting in a discontinuation of its development.

### 3.5. Anti-Tissue Factor-Based ADCs

Tissue factor (TF) is a well-known extrinsic coagulation factor with aberrant expression in many solid tumors including epithelial ovarian cancer, and it is implicated with neo-angiogenesis and cancer proliferation [54,55]. Tisotumab vedotin (TV, Seattle Genetics Inc., Bothell, WA, USA /Genmab, Copenhagen, Denmark) uses a protease-cleavable valine-citrulline linker to conjugate MMAE. The phase 1 InnovaTV201 trial has shown only modest activity in ovarian cancer patients (ORR 13.9%) [22]. A phase 2 trial (InnovaTV208, ClinicalTrials.gov Identifier: NCT03657043) in platinum-resistant ovarian carcinoma is currently ongoing.

### 3.6. Other Antigen-Based ADCs

The activity of other ADCs that target antigens overexpressed in ovarian cancer is under investigation in preclinical studies and early phase 1 trials. Protein kinase 7 (PTK7), involved in the Wnt pathway, T-cell immunoglobulin and mucin domain 1 (TIM1), Trophoblast cell-surface antigen 2 (TROP 2), and Notch 3 are a few examples of ovarian carcinoma antigens used in ADC development [23,24,25,26]. In the next few years, we will aim for an increased number of ADCs in the development in ovarian cancer and other solid tumors. The toxicity profile along with the high selectivity in delivering the cytotoxic payload makes this strategy an attractive approach for cancer treatment. PROBODY drug conjugates, molecules engineered with peptide masks that block normal tissue binding, can help to minimize on-target toxicity when the antigen is widely expressed in normal tissues [56]. In this regard, preliminary data on CX2009, a PROBODY drug conjugate targeting CD166, have been communicated in ASCO2020, with 2 PR in ovarian cancer patients [27].

## 4. Mechanisms of Resistance to ADCs

**Figure 1:**
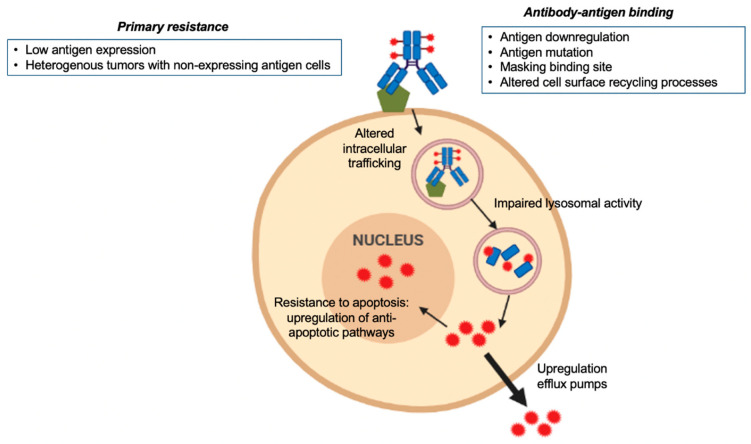
Mechanisms of resistance to antibody-drug conjugates (ADCs).

Mechanisms of primary and acquired resistance to ADCs are complex and depend on the components of the drug (Figure 1). Antibody-antigen binding, the payload type, and the chemical structure and stability of the linker are crucial steps for resistance to ADCs [57]. Heterogeneous tumors with a high proportion of antigen non-expressing tumor cells, or low expression of the targeted antigen, can diminish ADC efficacy and contribute to primary resistance to the drug. In addition, although it is not considered a resistance mechanism per se, premature payload deconjugation before reaching the tumor (i.e., cleavable linkers with unstable chemical structure) can decrease the final amount of ADC reaching the targeted tumor cells. The antibody-antigen binding process and the regulation of the surface antigen itself are key factors to induce acquired resistance [58]. Mutations in the expressed antigen, alterations in the cell surface recycling process along with downregulation of the antigen itself can alter antibody-antigen binding [59,60]. Other variations concerning the internalization process of the complex antibody-antigen or in the intracellular trafficking mechanism together with impaired lysosomal activity have also been described as possible mechanisms of acquired resistance [61,62,63]. Commonly shared with other cytotoxic agents, once the payload has been released into the target cell, efflux pumps can contribute to expelling the drug outside the cell. The upregulation of these pumps and systems is one of the main mechanisms of resistance described associated with cytotoxic agents and also described with ADCs [58,64].

## 5. Future Directions

While a great number of ADCs have been developed, only a minority have been approved in the last 20 years, and results in ovarian cancer, although promising, have not had a real clinical impact. Patient selection according to antigen expression is advisable in order to better identify the population who will most benefit from these therapies, and clinical translational research and bioinformatic approaches will be essential to identify biomarkers of response. Chemical modifications of ADC structure (i.e., peptide masking, increasing DAR, antibody fragments to increase ADC delivery to the tumor) will help to increase efficacy without increasing toxicity and will help to overcome resistance.

In addition, a certain subgroup of patients could have better outcomes with ADCs, such as the *BRCA* mutated population, in which a high therapeutic index of DNA damaging agents could result in greater responses. In this regard, the use of pyrrolobenzodiazepine dimers (PBD) as cytotoxic payload, which induce DNA double-strand breaks, could enhance ADCs’ efficacy in homologous recombination defective tumors [65]. Preliminary data on ADCs in combination with chemotherapy and targeted therapies have shown encouraging outcomes. Among them, combinations with immunotherapy need to be explored as immunogenic cell death driven by ADCs can enhance the efficacy of immune checkpoint inhibitors and other immune modulators [66,67].

## 6. Conclusions

ADCs can selectively deliver a cytotoxic agent intracellularly using a specific antibody-antigen binding. Their toxicity profile in comparison to classical chemotherapeutic agents makes ADCs an attractive strategy for cancer treatment. Ovarian cancer differentially expresses tumor-specific antigens, which makes this cancer type a good candidate for the development of ADCs. Results from early phase 1–2 trials differ between compounds, probably due to the percentage and intensity of antigen expression and the chemical structure of each ADC. To date, only one ADC, mirvetuximab soravtansine, has achieved phase 3 development, with modest results as a monotherapy treatment. However, preliminary data on combinations with chemotherapy and targeted therapies have shown encouraging activity. ADCs’ toxicity seems to be favorable with non-severe, reversible, and easily manageable off-target side effects. Mechanisms of resistance are diverse and complex and depend on the antibody-antigen binding process, the chemical structure of the linker and conjugation process, and the payload itself. Exploring the activity of ADCs in BRCA mutated and HR defective tumors and in combination with immunotherapy would be essential for further development of these agents.

## Figures and Tables

**Table 1:** Antigens and cytotoxic payloads for antibody-drug conjugates (ADCs) in clinical and preclinical development in ovarian cancer.

ADC	Antigen	Payload	Class	Mechanism of Action	Linker and DAR	Development Stage
Mirvetuximab soravtansine [13,14,15,16,17,18]	Folate receptor alpha	DM4	Maytansinoid	Microtubule-disrupting agent	Cleavable 3–4	Phase 3
Lifastuzumab vedotin [19]	NaPi2B	monomethyl auristatin E (MMAE)	Auristatin analogs	Microtubule-disrupting agent	Cleavable 3–4	Phase 2 (discontinued)
Sofituzumab vedotin [20]	MUC16	monomethyl auristatin E (MMAE)	Auristatin analogs	Microtubule-disrupting agent	Cleavable 3.5	Phase 1 (discontinued)
Anetumab ravtansine [21]	Mesothelin	DM4	Maytansinoid	Microtubule-disrupting agent	Cleavable 3.2	Phase 2
Tisotumab vedotin [22]	Tissue factor	monomethyl auristatin E (MMAE)	Auristatin analogs	Microtubule-disrupting agent	Cleavable NR	Phase 2
Cofituzumab pelidotin [23]	PTK7	Aur0101	Auristatin analogs	Microtubule-disrupting agent	Cleavable NR	Preclinical
CDX-014 [24]	TIM1	monomethyl auristatin E (MMAE)	Auristatin analogs	Microtubule-disrupting agent	Cleavable 4.5	Preclinical
Sacituzumab govitecan [25]	TROP-2	SN-38	Camptothecin	Topoisomerase inhibitor analog	Cleavable 6.78	Preclinical
PF-06650808 [26]	NOTCH-3	monomethyl auristatin E (MMAE)	Auristatin analogs	Microtubule-disrupting agent	Cleavable NR	Phase 1
Praluzatamab ravtansine, CX-2009 [27]	CD166	DM4	Maytansinoid	Microtubule-disrupting agent	Cleavable 3.5	Phase 1

DAR Drug-to-antibody ratio; NR Not reported.

**Table 2:** Main characteristics of ADC antigens for treating ovarian cancer.

Antigen	Function	Expression in Normal Cells	Expression in Ovarian Cancer Cells	ADC	Payload
FRα	Intracellular transport of folate	Marginally expressed in normal cells (polarized epithelium)	67–100%	Mirvetuximab soravtansine (IMGN853)	DM4
Mesothelin	Cell adhesion	Expressed in pleura, peritoneum and pericardium	55–100%	Anetumab ravtansine	DM4
Tissue factor	Extrinsic pathway of the coagulation cascade	Subendothelial vessel wall cells	23–100%	Tisotumab vedotin	MMAE
MUC16	Protection of epithelial surfaces	Epithelial cells (eye, mesothelium, trachea)	70–90%	Sofituzumab vedotin	MMAE
TROP2	Intracellular calcium signal transducer	Trophoblast cells, alveolar epithelial cells, smooth muscle cells	82–92%	Sacituzumab govitecan	SN-38
NaPi2B	Sodium-dependent surface transporter	Epithelial cells (pneumocytes, small bowel, mammary gland)	80–93%	Lifastuzumab vedotin (LIFA)	MMAE

**Table 3:** Clinical efficacy of ADCs in ovarian cancer.

ADCs	Target Antigen	Phase of Development	Efficacy of Monotherapy	Efficacy in Combination	Main Toxicity (>20%)
Mirvetuximab soravtansine [13,14,15,16,17,18]	FRα	Phase III	ORR 24–46% mPFS 4.8–6.7 months	Bev (platinum resistant): ORR 39% Carbo AUC4–5 (platinum sensitive): ORR 71%, mPFS 15 months	Ocular toxicity (blurred vision, keratopathy), neurotoxicity, fatigue, AST increased, nausea
STRO-002 [42]	FRα	Phase 1	ongoing	–	Fatigue, vomiting, decreased appetite, constipation, AST increased, neuropathy
MORAb-202 [43]	FRα	Phase 1	DCR 75% (1/9 CR; 2/9 PR)	–	ALT and GGT increased, leukopenia, neutropenia
Anetumab ravtansine [21]	Mesothelin	Phase 1b–2	ORR 9% DCR 59%	PLD: DCR 83% (52% PR, 33% SD)	Keratitis and neuropathy (both DLT). GI disorders
DMOT4039A [44]	Mesothelin	Phase 1	ORR 30% mPFS4.9 months	–	Diarrhea, nausea, fatigue, alopecia
Tisotumab vedotin [22]	Tissue factor	Phase 1–2	ORR 13.9%	–	Ocular toxicity (conjunctivitis, dry eye), epistaxis, fatigue, neuropathy, nausea, diarrhea, decreased appetite
Sofituzumab vedotin [20]	MUC16	Phase 1	ORR 17%	–	Fatigue, neuropathy, nausea, decreased appetite, diarrhea, alopecia, pyrexia, anemia, neutropenia, hypomagnesemia
DMU4C064A [45]	MUC16	Phase 1	ORR 45% (1CR/8 PR) mPFS 5.8 months	–	Ocular toxicity (visual disturbance, keratitis, dry eye), neuropathy, diarrhea, nausea, fatigue
Lifastuzumab vedotin (LIFA) [19]	NaPi2B	Phase 2	ORR 34% vs.15% (*p* = 0.03) mPFS 5.3 vs. 3.1 months (HR 0.71)	–	Neuropathy, diarrhea, nausea, constipation, neutropenia, anemia, fatigue
XMT1536 [46]	NaPi2B	Phase 1	2 CR, 11 prolonged SD	–	Nausea, fatigue, headache

FRα—folate receptor alpha; Bev—bevacizumab; AUC—area under the curve; PLD—pegylated lysosomal doxorubicin; ORR—overall response rate; mPFS—median progression-free survival; DCR—disease control rate; CR—complete response; PR—partial response; SD—stable disease.
